# Supplementary material for: Prognostic Significance of Cuproptosis-Related Gene Signatures in Breast Cancer Based on Transcriptomic Data Analysis
Source: Cancers (Basel). 2022 Nov 24;14(23):5771. doi: 10.3390/cancers14235771 (PMC9737541; doi:10.3390/cancers14235771)
Supplement: Supplementary file 1 [file cancers-14-05771-s001.zip › cancers-1993329-supplementary.pdf]

Title: Prognostic Significance of Cuproptosis-Related Gene Signatures in Breast Cancer Based on Transcriptomic Data Analysis

Figure legend for Supplementary figures:

Figure S1. Workflow diagram. The specific workflow graph of data analysis.

Figure S2. Identification of cancer patient subclusters based on the CRGs. (A) The relative change in area under consensus CDF curve for k=2 to 9. (B-E) The consensus clustering of BRCA patients (k was set as 6-9).

Figure S3. Comparison of the AUC between our risk model and other gene models. (A-B) The 1-year ROC curve analysis between our risk model and Jia Li et al., (A) and wei Li et al., (B) gene risk models. (C-D) The 3-year ROC curve analysis between our risk model and Jia Li et al.(C) and wei Li et al.(D) gene risk models. (E-F) The 5-year ROC curve analysis between our risk model and Jia Li et al. (E) and wei Li et al. (F) gene risk models.

Figure S4. Molecular biological functional analysis based on the DEGs among the two-risk groups in the TCGA cohort. (A-B) Bar plot for KEGG pathways of high-risk group (A) and low risk group (B). (C-D) Bubble plot for GO enrichment of high-risk group (C) and low risk group (D).

Figure S5. Comparison of the relationship between immune cells and risk score. (A) The relationship between immune cells and immune pathways. (B-N) the relationship between immune cells and risk score.

Figure S6. Comparison of the relationship between immune pathways and risk score. (A-P) The relationship between immune cells and risk score.

Figure S7. Screening independent prognostic factor and construction of clinical nomogram model. (A) Univariate analysis of indicators in TCGA cohort. (B) Multivariate analysis of indicators in TCGA cohort. (C) Univariate analysis of indicators in GEO cohort. (D) Multivariate analysis of indicators in GEO cohort. (E) Nomogram construction based on clinical indicators and risk score. (F) The calibration curves of nomogram at 1, 3 and 5 years.

Figure S8. Comparison of AUC and DCA between our nomogram model and TNM model. (A, D) 1-year AUC and DCA values. (B,E) 3-year AUC and DCA values . (C,F) 5-year AUC and DCA values.

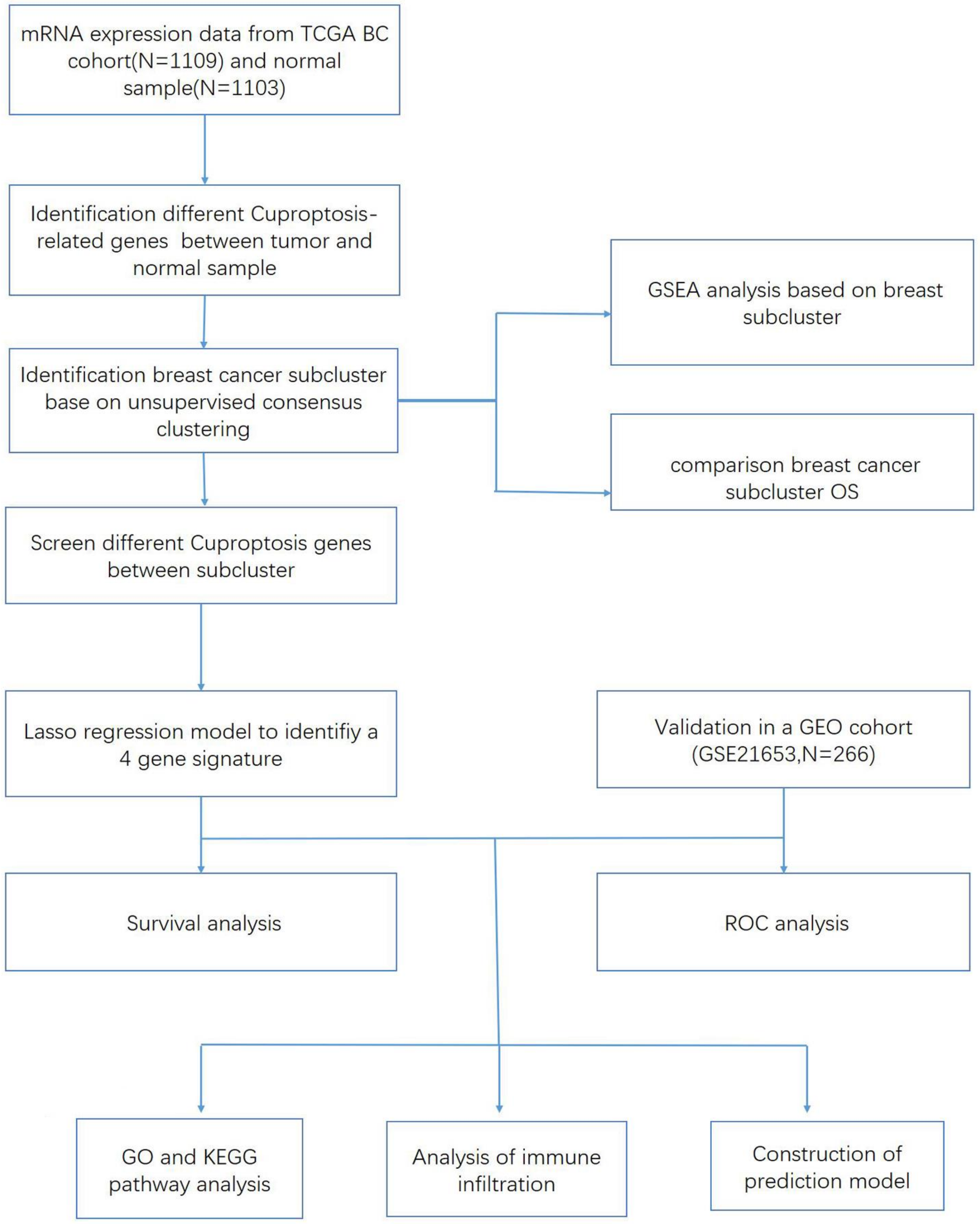
mRNA expression data from TCGA BC cohort(N=1109) and normal sample(N=1103)
Identification different Cuproptosis-related genes between tumor and normal sample
Identification breast cancer subcluster base on unsupervised consensus clustering
GSEA analysis based on breast subcluster
comparison breast cancer subcluster OS
Screen different Cuproptosis genes between subcluster
Lasso regression model to identifiy a 4 gene signature
Validation in a GEO cohort (GSE21653,N=266)
Survival analysis
ROC analysis
GO and KEGG pathway analysis
Analysis of immune infiltration
Construction of prediction model

Figure S1

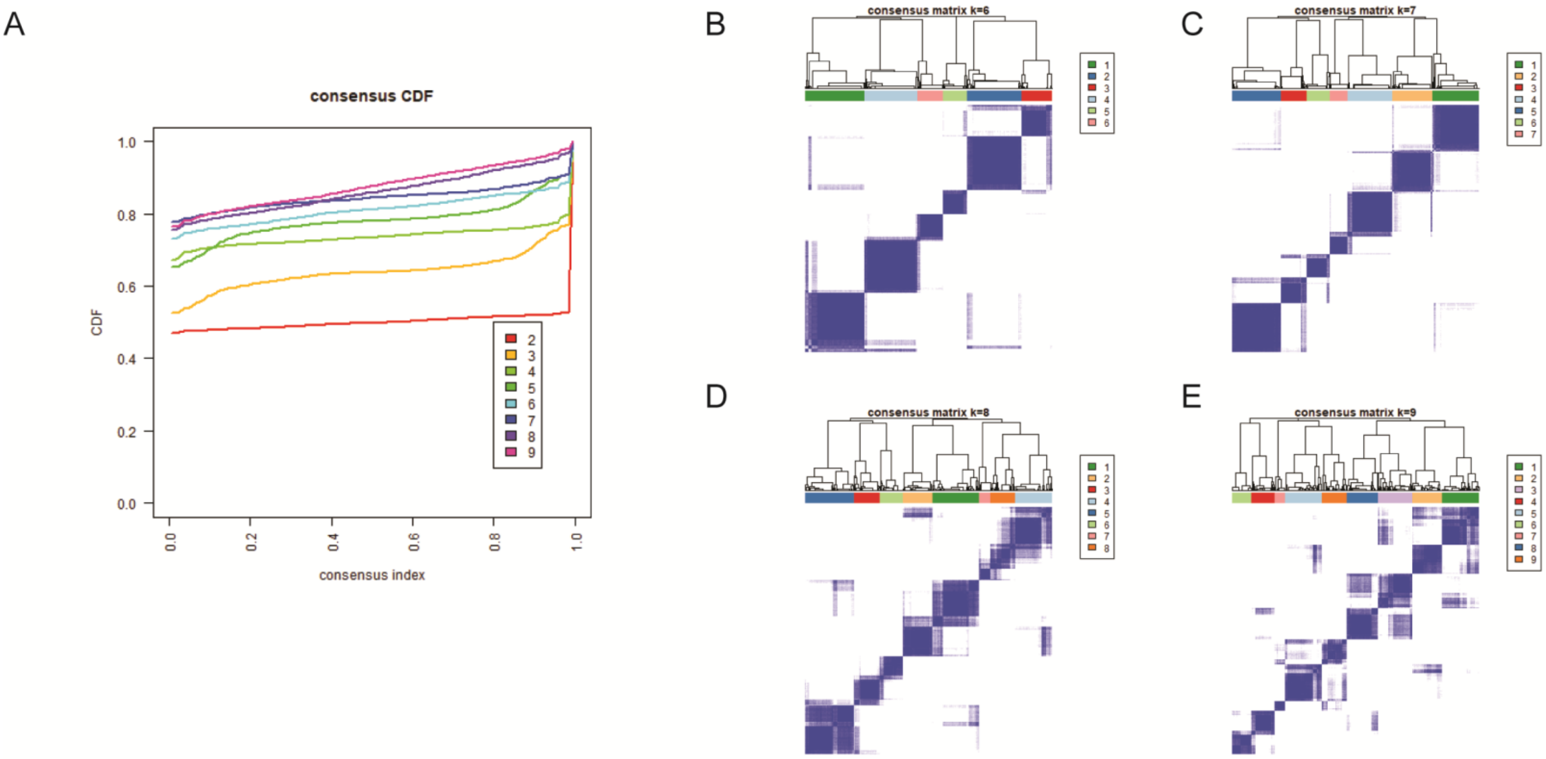
A
consensus CDF
CDF
consensus index
1.0
0.8
0.6
0.4
0.2
0.0
2
3
4
5
6
7
8
9
B
consensus matrix k=6
C
consensus matrix k=7
D
consensus matrix k=8
E
consensus matrix k=9

Figure S2

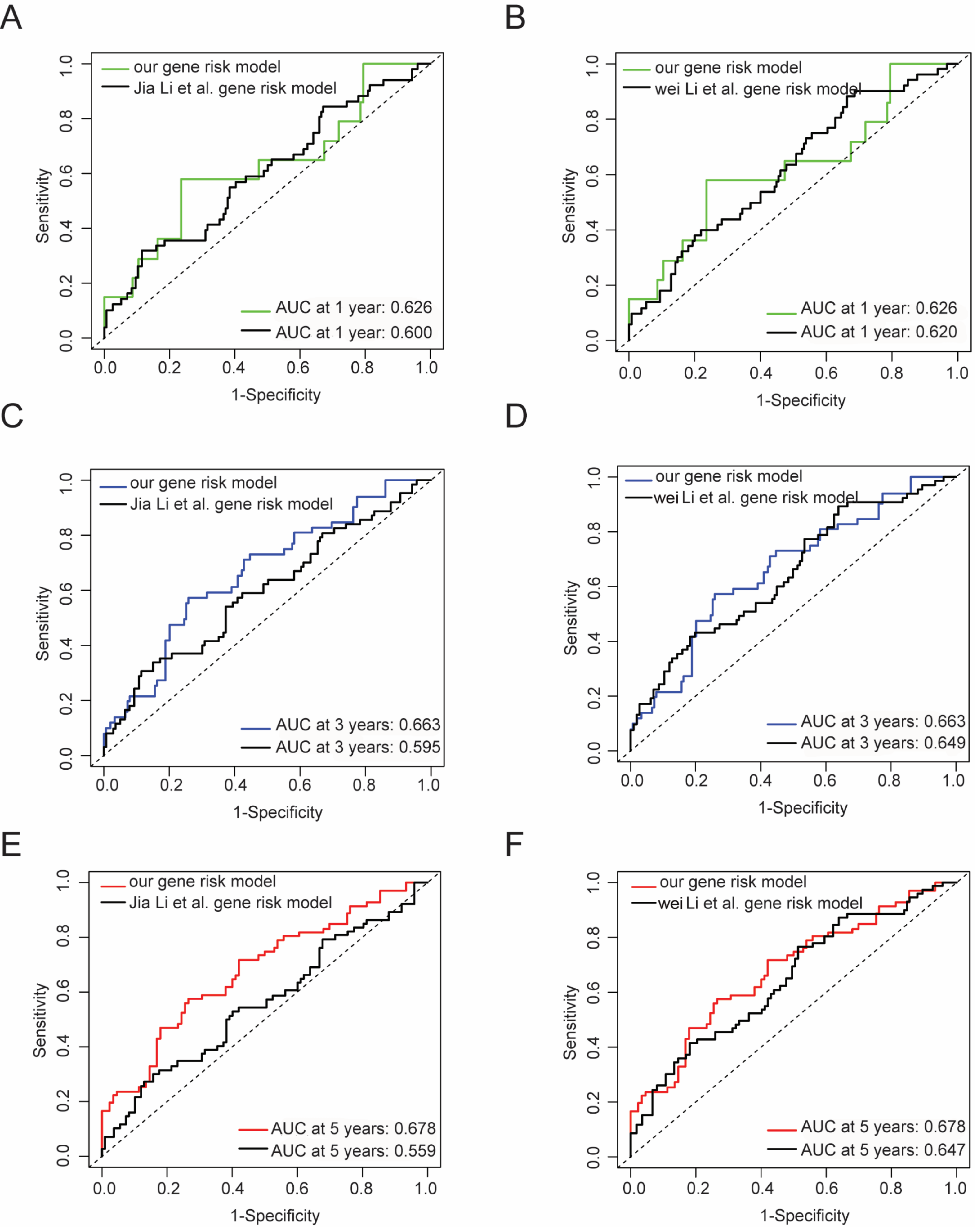
A
our gene risk model
Jia Li et al. gene risk model
AUC at 1 year: 0.626
AUC at 1 year: 0.600
Sensitivity
1-Specificity
B
our gene risk model
wei Li et al. gene risk model
AUC at 1 year: 0.626
AUC at 1 year: 0.620
Sensitivity
1-Specificity
C
our gene risk model
Jia Li et al. gene risk model
AUC at 3 years: 0.663
AUC at 3 years: 0.595
Sensitivity
1-Specificity
D
our gene risk model
wei Li et al. gene risk model
AUC at 3 years: 0.663
AUC at 3 years: 0.649
Sensitivity
1-Specificity
E
our gene risk model
Jia Li et al. gene risk model
AUC at 5 years: 0.678
AUC at 5 years: 0.559
Sensitivity
1-Specificity
F
our gene risk model
wei Li et al. gene risk model
AUC at 5 years: 0.678
AUC at 5 years: 0.647
Sensitivity
1-Specificity

Figure S3

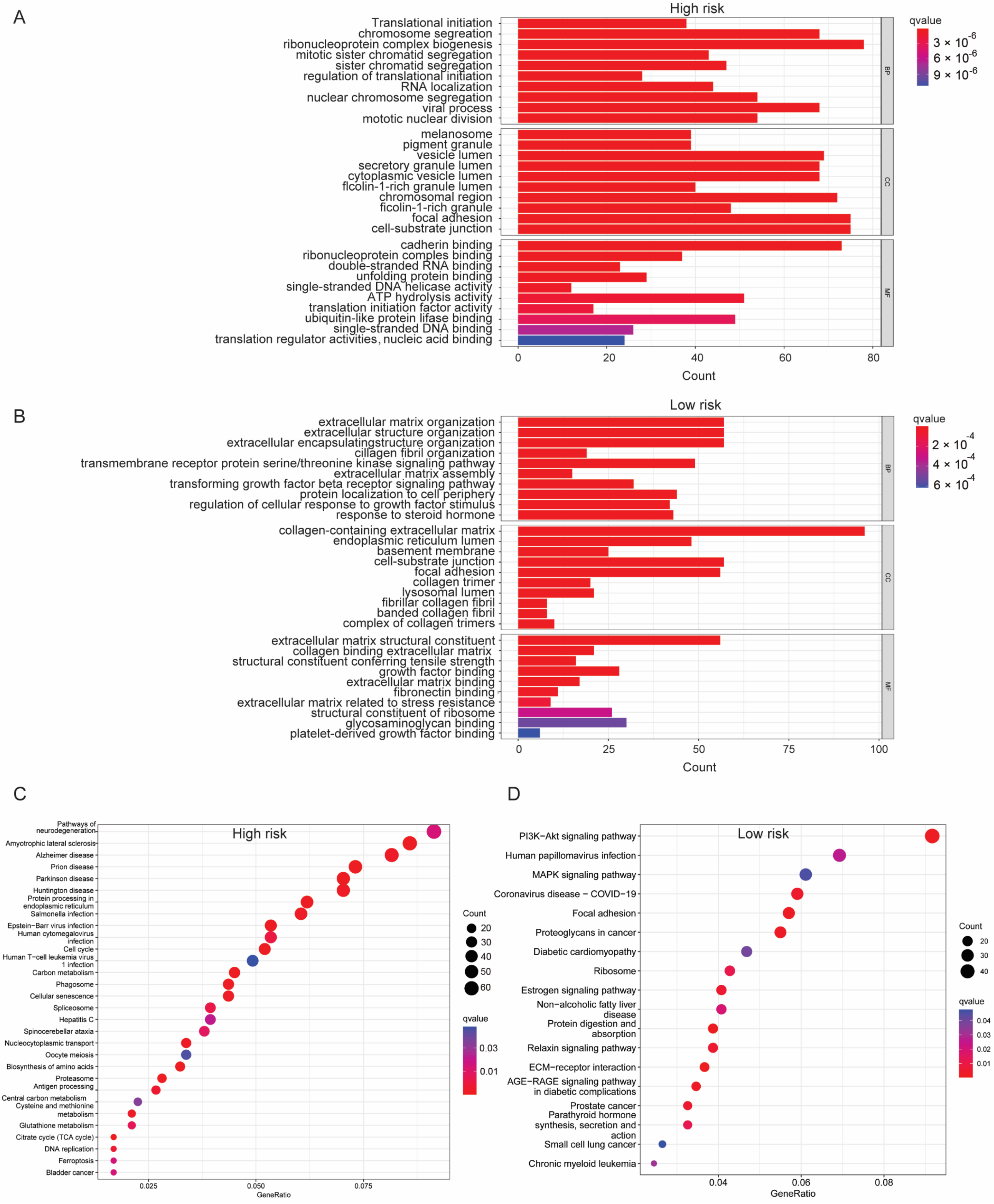
A
High risk
Translational initiation
chromosome segregation
ribonucleoprotein complex biogenesis
mitotic sister chromatid segregation
sister chromatid segregation
regulation of translational initiation
RNA localization
nuclear chromosome segregation
viral process
mototic nuclear division
melanosome
pigment granule
vesicle lumen
secretory granule lumen
cytoplasmic vesicle lumen
flcolin-1-rich granule lumen
chromosomal region
ficolin-1-rich granule
focal adhesion
cell-substrate junction
cadherin binding
ribonucleoprotein comples binding
double-stranded RNA binding
unfolding protein binding
single-stranded DNA helicase activity
ATP hydrolysis activity
translation initiation factor activity
ubiquitin-like protein lifase binding
single-stranded DNA binding
translation regulator activities, nucleic acid binding
BP
CC
MF
0
20
40
60
80
Count
qvalue
3 × 10^-6
6 × 10^-6
9 × 10^-6
B
Low risk
extracellular matrix organization
extracellular structure organization
extracellular encapsulatingstructure organization
cillagen fibril organization
transmembrane receptor protein serine/threonine kinase signaling pathway
extracellular matrix assembly
transforming growth factor beta receptor signaling pathway
protein localization to cell periphery
regulation of cellular response to growth factor stimulus
response to steroid hormone
collagen-containing extracellular matrix
endoplasmic reticulum lumen
basement membrane
cell-substrate junction
focal adhesion
collagen trimer
lysosomal lumen
fibrillar collagen fibril
banded collagen fibril
complex of collagen trimers
extracellular matrix structural constituent
collagen binding extracellular matrix
structural constituent conferring tensile strength
growth factor binding
extracellular matrix binding
fibronectin binding
extracellular matrix related to stress resistance
structural constituent of ribosome
glycosaminoglycan binding
platelet-derived growth factor binding
BP
CC
MF
0
25
50
75
100
Count
qvalue
2 × 10^-4
4 × 10^-4
6 × 10^-4
C
High risk
Pathways of neurodegeneration
Amyotrophic lateral sclerosis
Alzheimer disease
Prion disease
Parkinson disease
Huntington disease
Protein processing in endoplasmic reticulum
Salmonella infection
Epstein−Barr virus infection
Human cytomegalovirus infection
Cell cycle
Human T−cell leukemia virus 1 infection
Carbon metabolism
Phagosome
Cellular senescence
Spliceosome
Hepatitis C
Spinocerebellar ataxia
Nucleocytoplasmic transport
Oocyte meiosis
Biosynthesis of amino acids
Proteasome
Antigen processing
Central carbon metabolism
Cysteine and methionine metabolism
Glutathione metabolism
Citrate cycle (TCA cycle)
DNA replication
Ferroptosis
Bladder cancer
0.025
0.050
0.075
GeneRatio
Count
20
30
40
50
60
qvalue
0.03
0.01
D
Low risk
PI3K−Akt signaling pathway
Human papillomavirus infection
MAPK signaling pathway
Coronavirus disease − COVID−19
Focal adhesion
Proteoglycans in cancer
Diabetic cardiomyopathy
Ribosome
Estrogen signaling pathway
Non−alcoholic fatty liver disease
Protein digestion and absorption
Relaxin signaling pathway
ECM−receptor interaction
AGE−RAGE signaling pathway in diabetic complications
Prostate cancer
Parathyroid hormone synthesis, secretion and action
Small cell lung cancer
Chronic myeloid leukemia
0.04
0.06
0.08
GeneRatio
Count
20
30
40
qvalue
0.04
0.03
0.02
0.01

Figure S4

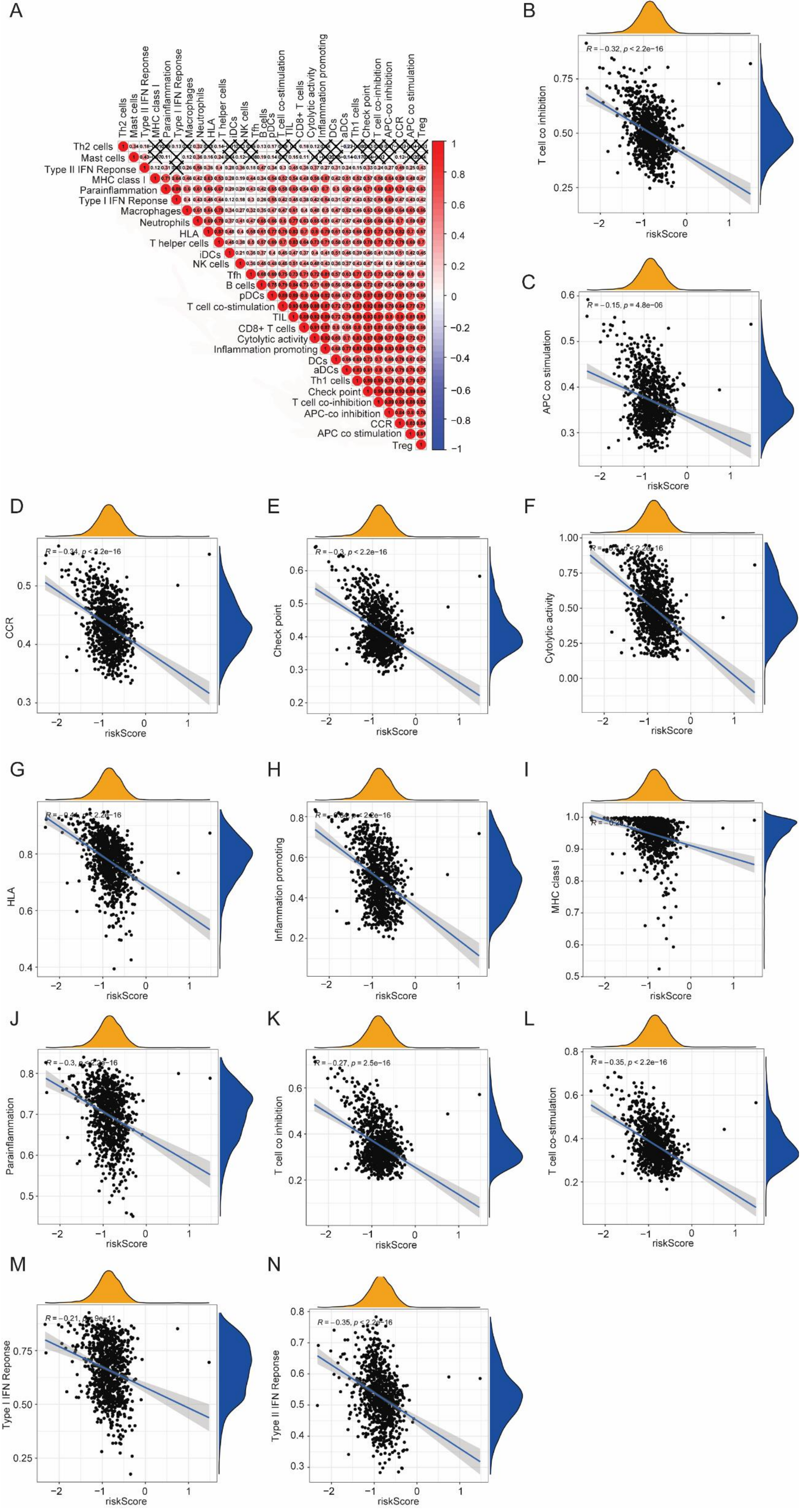
A
Th2 cells
Mast cells
Type II IFN Reponse
MHC class I
Parainflammation
Type I IFN Reponse
Macrophages
Neutrophils
HLA
T helper cells
iDCs
NK cells
Tfh
B cells
pDCs
T cell co-stimulation
TIL
CD8+ T cells
Cytolytic activity
Inflammation promoting
DCs
aDCs
Th1 cells
Check point
T cell co-inhibition
APC-co inhibition
CCR
APC co stimulation
Treg
B
R = −0.32, p < 2.2e−16
T cell co inhibition
riskScore
C
R = −0.15, p = 4.8e−06
APC co stimulation
riskScore
D
R = −0.34, p < 2.2e−16
CCR
riskScore
E
R = −0.3, p < 2.2e−16
Check point
riskScore
F
Cytolytic activity
riskScore
G
HLA
riskScore
H
Inflammation promoting
riskScore
I
MHC class I
riskScore
J
R = −0.3, p < 2.2e−16
Parainflammation
riskScore
K
R = −0.27, p = 2.5e−16
T cell co inhibition
riskScore
L
R = −0.35, p < 2.2e−16
T cell co-stimulation
riskScore
M
Type I IFN Reponse
riskScore
N
R = −0.35, p < 2.2e−16
Type II IFN Reponse
riskScore

Figure S5

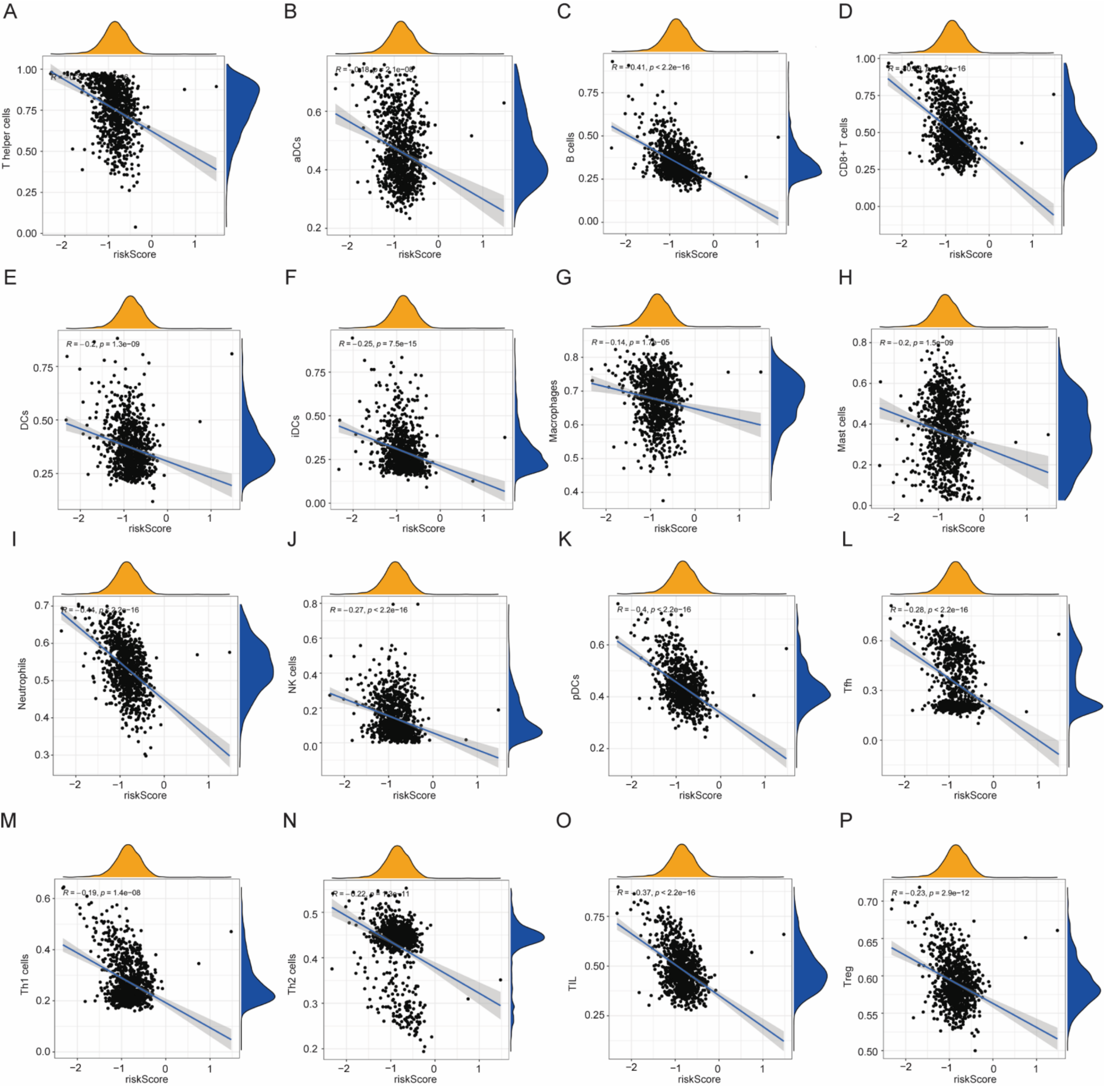
A
B
C
D
E
F
G
H
I
J
K
L
M
N
O
P
T helper cells
aDCs
B cells
CD8+ T cells
DCs
iDCs
Macrophages
Mast cells
Neutrophils
NK cells
pDCs
Tfh
Th1 cells
Th2 cells
TIL
Treg
riskScore
R = −0.41, p < 2.2e−16
R = −0.2, p = 1.3e−09
R = −0.25, p = 7.5e−15
R = −0.27, p < 2.2e−16
R = −0.4, p < 2.2e−16
R = −0.28, p < 2.2e−16
R = −0.19, p = 1.4e−08
R = −0.37, p < 2.2e−16
R = −0.23, p = 2.9e−12

Figure S6

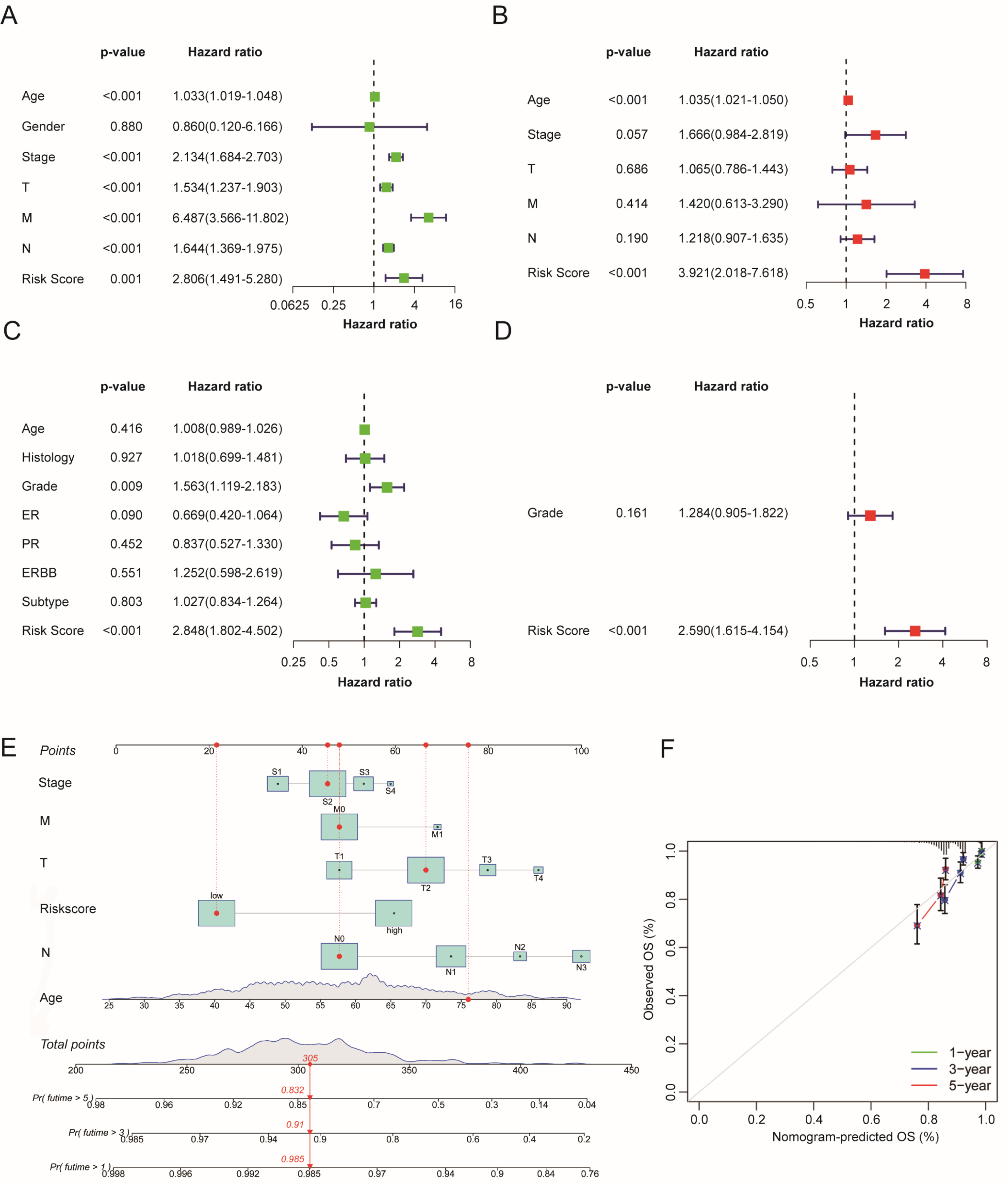
A
p-value Hazard ratio
Age <0.001 1.033(1.019-1.048)
Gender 0.880 0.860(0.120-6.166)
Stage <0.001 2.134(1.684-2.703)
T <0.001 1.534(1.237-1.903)
M <0.001 6.487(3.566-11.802)
N <0.001 1.644(1.369-1.975)
Risk Score 0.001 2.806(1.491-5.280)
Hazard ratio
B
p-value Hazard ratio
Age <0.001 1.035(1.021-1.050)
Stage 0.057 1.666(0.984-2.819)
T 0.686 1.065(0.786-1.443)
M 0.414 1.420(0.613-3.290)
N 0.190 1.218(0.907-1.635)
Risk Score <0.001 3.921(2.018-7.618)
Hazard ratio
C
p-value Hazard ratio
Age 0.416 1.008(0.989-1.026)
Histology 0.927 1.018(0.699-1.481)
Grade 0.009 1.563(1.119-2.183)
ER 0.090 0.669(0.420-1.064)
PR 0.452 0.837(0.527-1.330)
ERBB 0.551 1.252(0.598-2.619)
Subtype 0.803 1.027(0.834-1.264)
Risk Score <0.001 2.848(1.802-4.502)
Hazard ratio
D
p-value Hazard ratio
Grade 0.161 1.284(0.905-1.822)
Risk Score <0.001 2.590(1.615-4.154)
Hazard ratio
E
Points
Stage
M
T
Riskscore
N
Age
Total points
Pr(futime > 5)
Pr(futime > 3)
Pr(futime > 1)
F
Observed OS (%)
Nomogram-predicted OS (%)
1-year
3-year
5-year

Figure S7

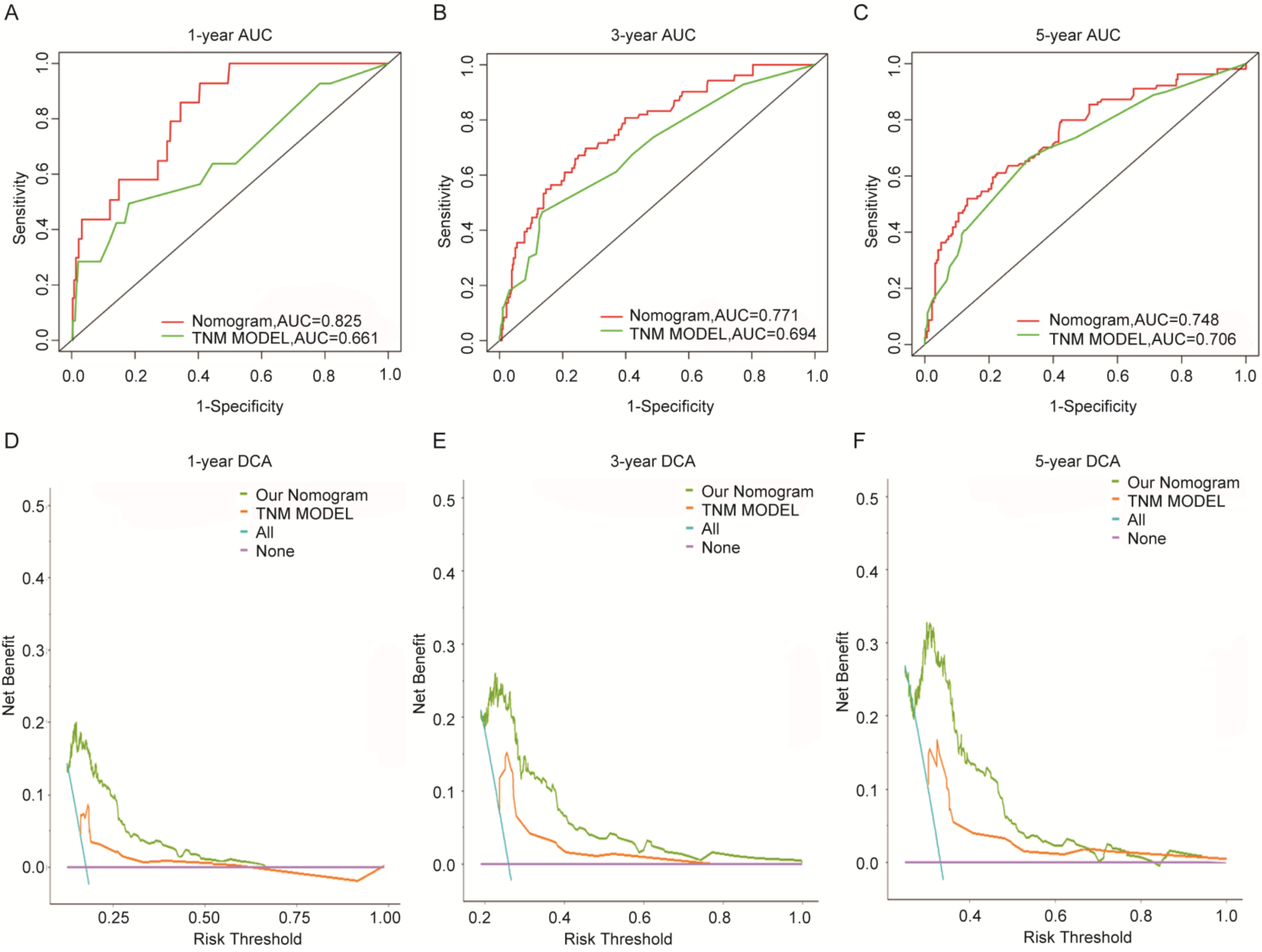
A
1-year AUC
Sensitivity
1-Specificity
Nomogram,AUC=0.825
TNM MODEL,AUC=0.661
B
3-year AUC
Sensitivity
1-Specificity
Nomogram,AUC=0.771
TNM MODEL,AUC=0.694
C
5-year AUC
Sensitivity
1-Specificity
Nomogram,AUC=0.748
TNM MODEL,AUC=0.706
D
1-year DCA
Net Benefit
Risk Threshold
Our Nomogram
TNM MODEL
All
None
E
3-year DCA
Net Benefit
Risk Threshold
Our Nomogram
TNM MODEL
All
None
F
5-year DCA
Net Benefit
Risk Threshold
Our Nomogram
TNM MODEL
All
None

Figure S8
